# Thrombin induces morphological and inflammatory astrocytic responses via activation of PAR1 receptor

**DOI:** 10.1038/s41420-022-00997-4

**Published:** 2022-04-11

**Authors:** Xiaojun Chen, Han Zhang, Huifei Hao, Xingyuan Zhang, Honghua Song, Bingqiang He, Yingjie Wang, Yue Zhou, Zhenjie Zhu, Yuming Hu, Yongjun Wang

**Affiliations:** 1 Key Laboratory of Neuroregeneration of Jiangsu and Ministry of Education, Co-innovation Center of Neuroregeneration, Nantong University, Nantong, 226001 PR China; 2 Department of Orthopaedics, Affiliated Hospital of Nantong University, Nantong University, Nantong, 226001 PR China; 3 Department of Rehabilitation Medicine, Affiliated Hospital of Nantong University, Nantong, 226001 PR China

**Keywords:** Cellular neuroscience, Astrocyte

## Abstract

Spinal cord injury (SCI) will result in the significant elevation of thrombin production at lesion site *via* either breakage of blood-spinal cord barrier or upregulated expression within nerve cells. Thrombin-induced activation of the protease activated receptors (PARs) evokes various pathological effects that deteriorate the functional outcomes of the injured cord. The cellular consequences of thrombin action on the astrocytes, as well as the underlying mechanism are not fully elucidated by far. In the present study, SCI model of rats was established by contusion, and primary astrocytes were isolated for culture from newborn rats. The expression levels of thrombin and PAR1 receptor at lesion sites of the spinal cord were determined. The primary astrocytes cultured in vitro were stimulated with different concentration of thrombin, and the resultant morphological changes, inflammatory astrocytic responses, as well as PAR1-activated signal pathway of astrocytes were accordingly examined using various agonists or antagonists of the receptor. Thrombin was found to reverse astrocytic stellation, promote proliferation but inhibit migration of astrocytes. Furthermore, the serine protease was shown to facilitate inflammatory response of astrocytes through regulation of MAPKs/NFκB pathway. Our results have provided the morphological evidence of astrocytic reactivity in response to thrombin stimulation and its neuroinflammatory effects following SCI, which will be indicative for the fundamental insights of thrombin-induced neuropathology.

## Introduction

Traumatic injury to the central nervous system (CNS) always results in the damage of the blood–brain/spinal cord barrier (BBB or BSCB), which triggers a series of cellular and molecular changes at lesion site that eventually impact on the functional outcomes [1–3]. Thrombin, a serine protease involved in haemostasis, is rapidly activated from blood-derived prothrombin by the combined actions of factors V and X in the presence of ionized calcium (Ca^2+^) [4–7], or sensitively induced within nerve cells [8]. High levels of thrombin have been shown to facilitate neurological injury by performance on neurotoxicity, vascular disruption, microglial activation, oligodendrogliopathy, and astrogliosis [9–14]. Selective blockade of thrombin signaling can significantly decrease neuronal death and improve functional recovery of injured or degenerative CNS diseases [8, 10, 15]. Thrombin-mediated cell events are achieved by the proteolytic activation of a family of G-protein-coupled receptors known as the protease activated receptors (PARs) [16–18]. The cleavage of the extracellular NH_2_ terminus of the PARs by the thrombin unmasks an amino acid sequence that acts as a tethered receptor ligand, thereby evokes multiple cellular responses in various nerve cells of CNS [16, 19–21].

Astrocytes are prominent participants of neuropathology following CNS injury. They undergo proliferation, morphologic changes, and cellular hypertrophy under the pathological conditions [22–25]. Once challenged by an inflammatory component or injury, the typical star-like astrocytes become hypertrophic and convert to flat morphology [26, 27]. The processes of the reactive astrocytes interacting with neurons change their morphology to cover a larger surface area of the neuronal soma [26]. Although the reactive astrocytes can form borders to restrict the entry of inflammatory cells into CNS parenchyma, they also have powerful pro-inflammatory potential that influences the functional recovery of the injured CNS [28–31]. The relevant factors of inducing reactive astrocytes in vivo are not fully elucidated, despite several cytokines and growth factors are found to be associated with such glial reaction [32]. Interestingly, activation of thrombin receptor after brain injury is able to trigger astrogliosis, and also generates cognitive deficit, suggesting that thrombin is an active player in mediating astrocytic reaction after CNS insult [10, 14]. However, the mechanism underlying the changes in morphology and effectors of astrocytes following the challenge of the thrombin remains unknown.

It has been known that a total of four subtypes of PARs, namely PAR1, PAR2, PAR3, and PAR4 are functionally co-expressed in the astrocytes [33]. Of them, PAR1, PAR3, and PAR4 are mainly activated by thrombin [33–35], while PAR2 is considered as typsin and mast cell tryptase receptor [36]. PAR1 serves as critical mediator in neuropathology following SCI by contributing to astrogliosis and augmentation of inflammation, and PAR1 knockout mice displayed a significant improvement of locomotor recovery [8]. As such, injury-induced production of thrombin may trigger astrocytic reaction via PAR1, thereby impacts on the neuropathological progression of the injured spinal cord. The mechanisms by which thrombin modulates cell events of astrocytes appear to be associated with the PAR-1 receptor signaling to activate TRPC3, a nonselective cation channel involved in Ca^2+^ influx, as well as its downstream MAPKs and Rho GTPase [14, 20, 26]. However, the dynamic regulation of thrombin on the intracellular signals of astrocytes is not fully elucidated by far. In the present study, we observed the cellular consequences of thrombin action on astrocytes isolated from rat spinal cord, and addressed the underlying mechanism. Our results will be beneficial for insights into the effects of SCI-activated thrombin on the astrocytic reaction and related neuropathology.

## Results

### Thrombin is inducibly activated following rat SCI

**Fig. 1:**
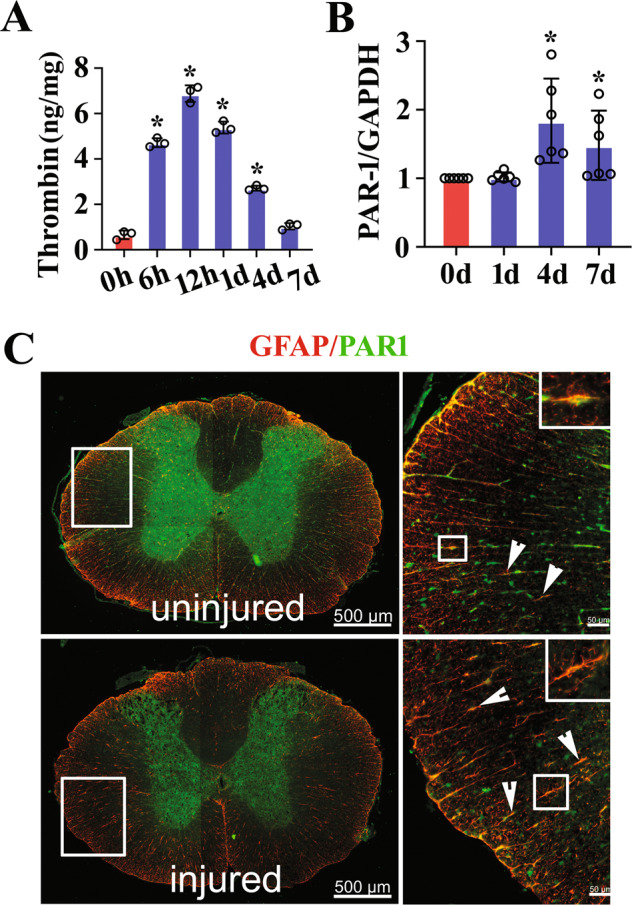
Expression analysis of thrombin and PAR1 receptor at lesion sites following SCI. **A** ELISA measurement of thrombin protein levels at lesion sites following SCI at 0 h, 6 h, 12 h, 1d, 4d, and 7d, respectively. **B** RT-PCR analysis of PAR1 expression following SCI at 0d, 1d, 4d, and 7d, respectively. Quantities were normalized to endogenous GAPDH. **C** Immunostaining showed colocalization of PAR1 with GFAP-positive astrocytes before or after SCI at 1d. Rectangle indicates region magnified. Arrowheads indicate positive signals. Experiments were performed in triplicates. Error bars represent the standard deviation (*P < 0.05). Scale bars, 500 μm in **C** and 50 μm in magnification.

To understand the effects of thrombin on the cellular consequences of astrocytes, the protein levels of thrombin at lesion sites of the spinal cord were determined by ELISA following contusion at 0 h, 6 h, 12 h, 1d, 4d, and 7d, respectively. Results demonstrated that SCI resulted in activation of thrombin within 4 days with a peak at 12 h (Fig. 1A). As PARs play a major role in thrombin-mediated cellular responses, and the prototype PAR1 is the most abundant family member in the CNS [37, 38], the dynamic profile of PAR1 expression was thus examined. RT-PCR results showed that PAR1 transcription was significantly upregulated at 4d and 7d following SCI (Fig. 1B). Further immunostaining of the cord sections before or after injury displayed that PAR1 (Alexa Fluor 488-labeled) colocalized with GFAP-positive (Cy3-labeled) astrocytes (Fig. 1C). The data indicate that injury-induced elevation of thrombin can potentially activate the PAR1 receptor of astrocytes at lesion site following SCI.

### Thrombin induces reversal of astrocyte stellation

**Fig. 2:**
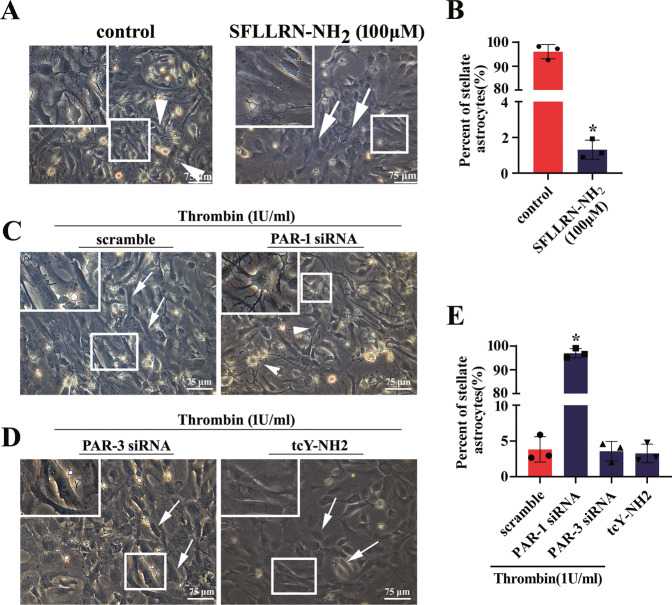
Effects of thrombin-mediated PAR1 activation on the morphological changes of astrocytes. **A** Observation of astrocyte morphological changes stimulated with 100 μM PAR1 agonist SFLLRN-NH_2_ for 2 h. **B** Statistical analysis of **A** in triplicates each 50 fields. **C**, **D** Astrocytes were transfected with PAR1 siRNA3 (**C**) and PAR3 siRNA1 (**D**) for 36 h prior to incubation with 1 U/ml thrombin for 2 h, or treated with 100 μM PAR4 specific inhibitor tcY-NH_2_ in the presence of 1 U/ml thrombin for 2 h (**D**). **E** Statistical analysis of **C** and **D** in triplicates each 50 fields. Arrowhead indicates stellate astrocyte, whereas arrow indicates fibroblast-like astrocyte. Rectangle indicates region magnified. Error bars represent the standard deviation (*P < 0.05). Scale bars, 75 μm.

Thrombin can evoke morphological changes of endothelial cells and fibroblasts [39, 40]. Also, it is found to induce the formation of expansive cell-free areas (CFAs) of astrocytes by morphological rearrangement in mixed rat hippocampal cultures [41]. To observe the effects of thrombin on the morphological changes of astrocytes cultured in serum-free medium, primary astrocytes with purity over 95% (Supplementary Fig. S1A) were stimulated with 0–10 U/ml thrombin for 2 h. PAR1 receptor was still detectable in the primary cells being shown by immunostaining (Supplementary Fig. S1B). When challenged with 0–10 U/ml thrombin, astrocytes under serum-free conditions lost their star shape, but flattened and acquired a fibroblast-like morphology (Supplementary Fig. S1C, D). MTT assay demonstrated that thrombin was able to enhance the viability of astrocytes, rather than elicited cytotoxicity (Fig. S2A). To address whether the thrombin-induced glial reactivity is associated with the activation of PAR1, 100 μM PAR1 agonist SFLLRN-NH_2_ was added to the culture for 2 h. A dramatic change of astrocyte morphology was seen to replicate that of thrombin action (Fig. 2A, B). Knockdown of PAR1 receptor with the efficient siRNA3 (Fig. S2C) in the presence of 1 U/ml thrombin reversed the effects of thrombin on the stellate morphology of astrocytes (Fig. 2C, E). Whereas inhibition of PAR3 by siRNA1 (Fig. S2D), or PAR4 by specific inhibitor tcY-NH_2_, displayed undetectable reversal effects induced by thrombin in comparison with the control (Fig. 2D, E). The data indicate that thrombin is able to induce reversal of astrocyte stellation through activation of PAR1 receptor.

### Thrombin promotes proliferation but inhibits the migration of astrocytes

**Fig. 3:**
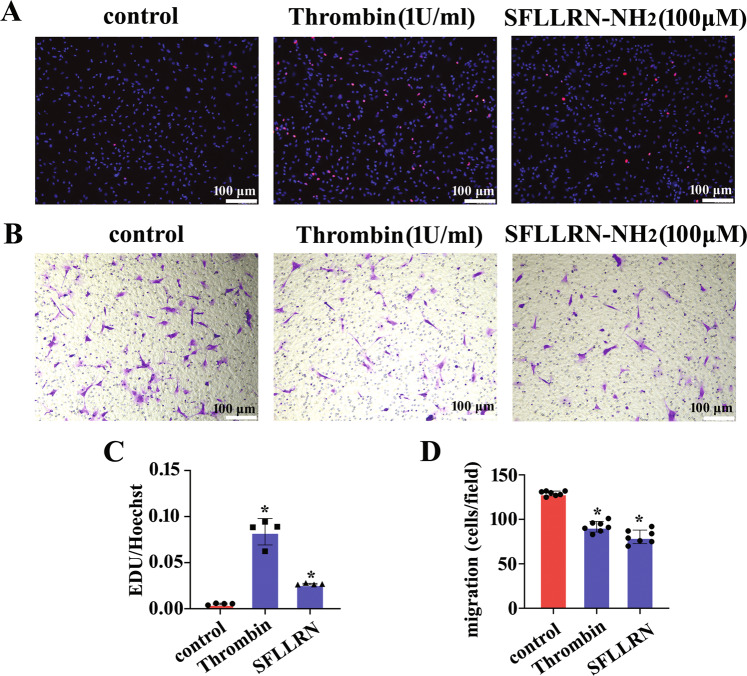
Thrombin promoted proliferation but inhibited migration of astrocytes. **A** EDU assay of astrocyte proliferation following stimulation with 1 U/ml thrombin or 100 μM PAR1 agonist SFLLRN-NH_2_ for 24 h. **B** Transwell assay of astrocyte migration following stimulation with 1 U/ml thrombin or 100 μM PAR1 agonist SFLLRN-NH_2_ for 24 h. The cells were stained with 0.1% crystal violet. **C**, **D** Quantification data as shown in **A** and **B**. Experiments were performed in triplicates. Error bars represent the standard deviation (*P < 0.05). Scale bars, 100 μm.

The number of reactive astrocytes and their secretions at lesion sites are tightly associated with the functional outcomes of the injured CNS [23, 25]. To observe the effects of thrombin on the proliferation and migration of astrocytes, primary astrocytes were incubated at 1 U/ml thrombin or 100 μM PAR1 agonist for 24 h under serum-free medium conditions. EDU assay displayed that both thrombin and the PAR1 agonist SFLLRN-NH_2_ were able to promote proliferation of the astrocytes (Fig. 3A, C). However, they significantly inhibited the cell migration comparing with the control (Fig. 3B, D). The data indicate that SCI-induced increase of thrombin is capable of promoting proliferation but negatively affecting the migration of astrocytes.

### Thrombin mediates inflammatory response of astrocytes

**Fig. 4:**
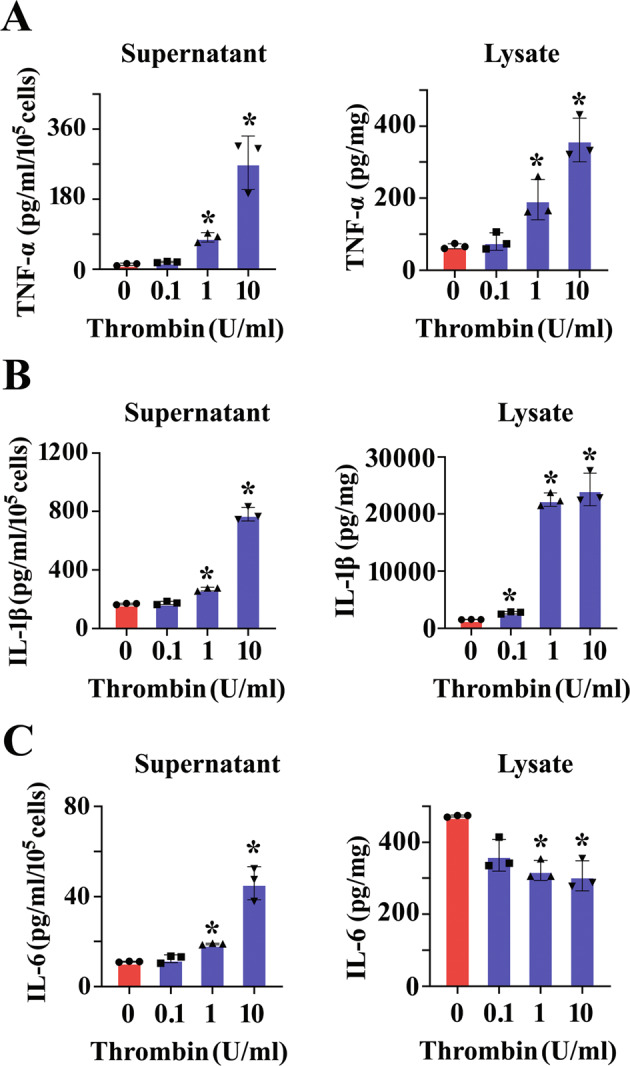
Thrombin-induced production of inflammatory cytokines from astrocytes. **A**–**C** ELISA assay of TNF-α (**A**), IL-1β (**B**), and IL-6 (**C**) in the supernatant and lysate of astrocytes following stimulation with 0–10 U/ml thrombin for 24 h. Experiments were performed in triplicates. Error bars represent the standard deviation (*P* < 0.05).

Reactive astrocytes are active contributors to neuropathology by potentiating innate immunity following CNS injury [25]. To gain insights into the function of thrombin in mediating inflammatory response of astrocytes, the cells were stimulated with 0-10 U/ml thrombin for 24 h. ELISA results showed that thrombin treatment of astrocytes at concentration over 1 U/ml was able to promote the production of TNF-α, IL-1β, and IL6 in the supernatant and lysate (Fig. 4A–C). However, the serine protease was incapable of facilitating the production of anti-inflammatory cytokines IL-4 and IL-10 (Supplementary Fig. S3A, B). The data indicate that thrombin is able to induce inflammatory astrocytic responses.

### Thrombin activates intracellular MAPKs pathway of astrocytes via PAR1 receptor

**Fig. 5:**
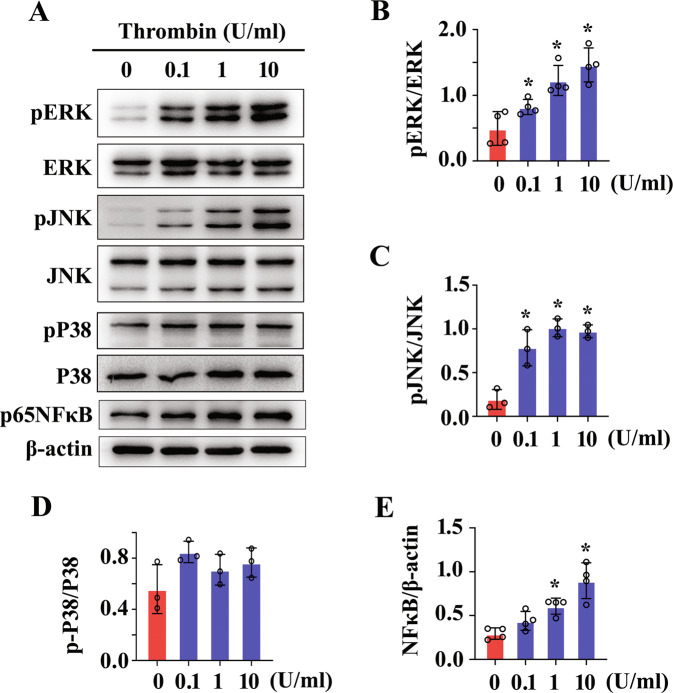
Determination for the activation of MAPKs/NFκB signaling in the astrocytes following stimulation with rat thrombin. **A** Western blot analysis of phosphorylated ERK, P38, JNK kinase, and NFκB protein after stimulation of astrocytes with 0–10 U/ml rat thrombin for 24 h. **B**–**E** Quantification data as shown in **A**. Quantities were normalized to endogenous β-actin. Experiments were performed in triplicates. Error bars represent the standard deviation (*P < 0.05).

**Fig. 6:**
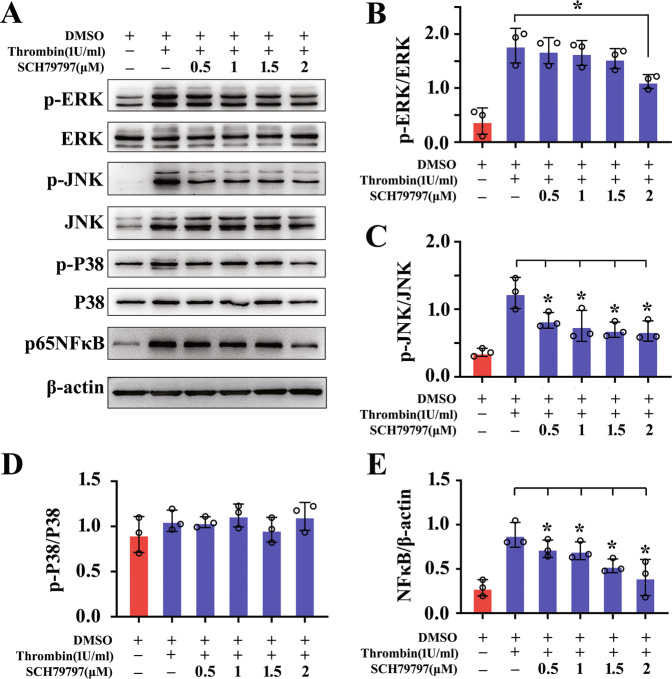
PAR1 inhibitor attenuated thrombin-induced activation of MAPKs/NFκB signaling in the astrocytes. **A** Western blot analysis of phosphorylated ERK, P38, JNK kinase, and NFκB protein after astrocyte treatment with 0–2 μM PAR1 inhibitor SCH79797 in the presence of 1 U/ml thrombin for 24 h. **B**–**E** Quantification data as shown in **A**. Quantities were normalized to endogenous β-actin. Experiments were performed in triplicates. Error bars represent the standard deviation (*P < 0.05).

Many of the cellular events occurring in reactive astrocytes are regulated by MAPKs/NFκB axis following CNS damage [31, 42]. To ascertain whether thrombin-mediated behaviors of astrocytes attribute to the activation of intracellular MAPKs/NFκB pathway, the phosphorylation of ERK, JNK, P38, and NFκB was determined following astrocyte treatment with 0–10 U/ml thrombin for 24 h. Results demonstrated that phosphorylated activation of ERK, JNK, and NFκB, rather than P38, was significantly increased in concentration dependence of thrombin (Fig. 5A–E). Addition of 100 μM PAR1 agonist SFLLRN-NH_2_ in the culture medium of astrocytes for 24 h exhibited consistent results with those of thrombin treatment (Supplementary Fig. S3C–G). Inhibition of PAR1 receptor with 0–2 μM SCH79797, the concentration was shown to have non-toxicity to the astrocytes (Fig. S2B), for 24 h in the presence of 1 U/ml thrombin resulted in a remarkable decrease of phosphorylated ERK, JNK, and NFκB protein, but not of P38, in concentration dependence of the inhibitor (Fig. 6A–E). The data indicate that thrombin is able to activate intracellular MAPKs pathway of astrocytes via PAR1 receptor.

### Blocking PAR1 receptor is able to attenuate thrombin-induced inflammatory response of astrocytes

**Fig. 7:**
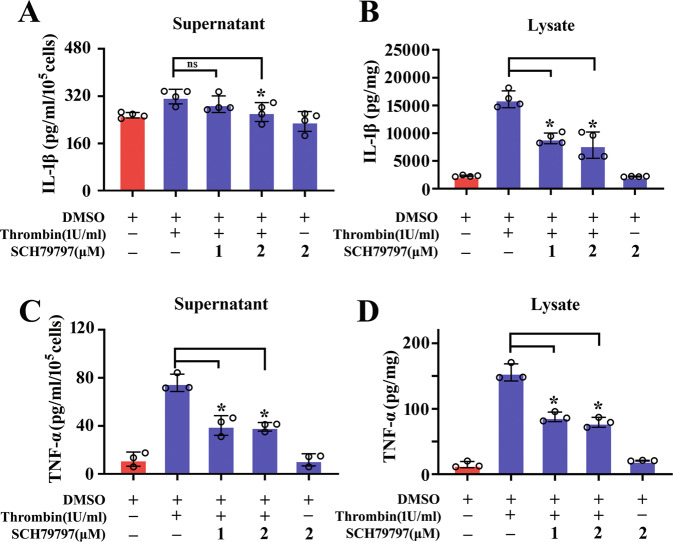
PAR1 inhibitor decreased thrombin-induced production of inflammatory cytokines. **A**–**D** ELISA assay of IL-1β (**A**, **B**) and TNF-α (**C**, **D**) in the supernatant and lysate of astrocytes following treatment with 0–2 μM PAR1 inhibitor SCH79797 in the presence of 1 U/ml thrombin for 24 h. Experiments were performed in triplicates. Error bars represent the standard deviation (*P < 0.05).

To understand the importance of PAR1 activation in thrombin-mediated inflammatory responses of astrocytes, 0–2 μM PAR1-specific inhibitor SCH79797 was added to the serum-free medium of astrocytes for 24 h in the presence of 1 U/ml thrombin. ELISA results displayed that the production of TNF-α and IL-1β was significantly attenuated by the inhibitor (Fig. 7A–D). The data indicate that inhibition of PAR1 receptor is able to reduce thrombin-induced production of inflammatory cytokines from astrocytes.

## Discussion

Thrombin is the activated product of prothrombin being generated in the CNS either immediately after breakdown of the BBB or BSCB, or by endogenous upregulation within nerve cells [10, 43, 44]. In addition to serving as a clotting factor, thrombin plays other multiple pathophysiological functions in the CNS. It regulates neural development and synaptic plasticity [21, 45]. Also, the serine protease has been shown to play neurotoxic roles [10, 46–50]. In the present study, we have demonstrated that thrombin is able to induce astrocyte reaction following SCI, which is associated with activation of neuroinflammation. Therefore, interference of the thrombin or its receptor has been found to be beneficial for reducing neuronal damage and improving neurobehavioral recovery following SCI or acute focal ischemia [8, 10], though considerable evidences indicate that low concentrations of thrombin are neuroprotective [43, 51]. Both neurons and astrocytes in the CNS contain all PAR receptors, and PAR1 activation is primarily responsible for the signal transduction of thrombin in the CNS [33, 52]. Thus, an immediate control of excessive thrombin activation or/and blocking of PAR1 receptor might be a promising strategy in alleviating thrombin-mediated neuropathological progression following SCI.

The protoplasmic astrocytes in the gray matter have short and largely branched tertiary processes, whereas fibrous astrocytes in the white matter exhibit long unbranched processes [23]. They act roles in supply of nutrients, regulation of neurotransmitters and maintenance of homeostasis of CNS [25]. Here, we showed that SCI resulted in elevation of thrombin protein levels, which was found to activate PAR1 receptor of astrocytes in vitro, and eventually gave rise to fibroblast-like morphological changes with contraction of processes. The loss of stellate morphology for the reactive astrocytes will inevitably reduce their interconnection and communications with other individual cells, blood capillaries, and synapses, contributing to a wide range of neuropathologies [23, 53]. These pathological features are always observed in ischemia, traumatic brain and spinal cord, as well as multiple neurodegenerative diseases [54]. Thrombin-induced morphological changes of astrocytes are involved in the activation of small GTP-binding proteins of the Ras superfamily, Rho, Rac, and Cdc42, because the injection of active Rho into stellate astrocytes mimicked the effect of thrombin [26]. In the present study, we also demonstrated that thrombin promoted proliferation but inhibited migration of astrocytes. It might be associated with the activation of proliferation-associated MAPKs pathway of astrocytes, but negative regulation of lamellipodia and filopodia under control of small GTP-binding proteins. However, the exact mechanism deserves further study.

Considerable evidence has shown that reactive astrocytes may exacerbate inflammatory responses and tissue damage following SCI, though they also participate in immunosuppression and tissue repair depending on timing [55]. We displayed that thrombin was able to promote the production of proinflammatory cytokines of astrocytes in concentration-dependent manner *via* activation of PAR1, thereby worsening inflammatory milieu. As an active player of the excessive inflammation, thrombin-induced astrocytes possibly act roles of inflammation-related neurotoxicity involved in the secondary degenerative process including the death of neurons and oligodendrocytes [15, 56]. Taken together, control of excessive thrombin activation following SCI may contribute to the mitigation of neuroinflammation and the resultant functional loss.

PLCε contains the X, Y, and C2 domains characteristic of enzymes in the phospholipase C (PLC) family [57], which is an important signaling node through which G-protein coupled receptors (GPCRs) activate small G-proteins to mediate biological responses [58]. PLCε is uniquely regulated by the small G-protein RhoA through a 65 amino acid sequence within the Y domain [59, 60], and by other Ras family members through their interactions with the RA2 domain [60]. Activation of PAR receptors has been found to significantly promote expression of PLCε, which is required for sustained activation of protein kinase D (PKD) and nuclear translocation of NFκB responsible for expression of inflammatory genes [58, 61]. In addition, PLCε has been found in association with the regulation of ERK signaling and DNA synthesis. As such, thrombin-induced inflammation and proliferation of astrocytes may be associated with the PAR1-mediated activation of PLCε, which in turn regulates MAPKs/NFκB pathway for the cell events.

In conclusion, SCI-induced elevation of thrombin at lesion sites can give rise to the activation of PAR1 receptor of astrocytes and thereby affects the astrocytic events. The serine protease is able to reverse astrocyte stellation and promote proliferation of the astrocytes. Also, it can mediate the proinflammatory response of the reactive astrocytes *via* activation of intracellular MAPKs/NFκB pathway. Inhibition of thrombin activity or the PAR1 receptor following SCI may contribute to alleviation of thrombin-mediated neuropathology.

## Materials and methods

### Animals

Adult male Sprague-Dawley (SD) rats, weighing 180–220 g, were supplied by the Center of Experimental Animals, Nantong University. All animal experiments were approved by the Animal Care and Use Committee of Nantong University and the Animal Care Ethics Committee of Jiangsu Province. All rats were housed in standard cages (five rats in each cage) in an environment controlled at temperature of 22 ± 2 °C with a 12–12 h light–dark cycle. The rats had free access to water and food.

### Establishment of contusion SCI rat model

The animals for the surgery were randomized and grouped at six per experimental group in triplicate. The contusion model of rat spinal cord was established as the previous description [62]. All animals were anesthetized with 10% chloral hydrate (300 mg/kg) administered intraperitoneally. After the shaved skin around the surgical site was disinfected with iodophor, the spinous processes of T8-T10 vertebrae were surgically exposed, and a dorsal laminectomy at T9 of the thoracic vertebra was performed. The exposed spinal cord segment at 3 mm length received a 150-kilodyne contusion injury using the IH-0400 Impactor (Precision Systems and Instrumentation) injury device, and then the incision was irrigated and sutured. The rats were subcutaneously administered with 0.2 ml antibiotics following surgery. Manual evacuation of urine was performed twice a day until the animal regained control of normal micturition.

### Cell culture and treatment

Astrocytes were isolated and cultured as previous description [63]. Briefly, the spinal cords of newborn SD rats, 1–2 days after birth, were removed from the spinal canal and placed into 0.01 M PBS containing 1% penicillin-streptomycin. Then the spinal cords were minced with scissors and digested with 0.25% trypsin for 15 min at 37 °C. Enzymatic digestion of tissue was terminated by Dulbecco’s Modified Eagle’s Medium - high glucose medium containing 10% fetal bovine serum (FBS), 1% penicillin-streptomycin and 1% l-glutamine. The suspension was centrifuged at 1200 rpm for 5 min, and the cells were resuspended and seeded onto poly-l-lysine pre-coated culture flask in the presence of 5% CO_2_. After cell culture for 7–9 days with medium replacement every 3 days, the non-astrocytes were removed by shaking culture flask at 250 rpm overnight. The phenotype of the isolated astrocytes was evaluated by the characteristic morphology and immunostaing of the astrocytic marker glial fibrillary acid protein (GFAP). Astrocytes with purity more than 95% were allowed for the desired experiments.

To examine the effects of thrombin on astrocytes, the serum was removed by washing 3 times during 15 min in serum-free DMEM:F-12 medium. The cells were then subjected for 2 h or 24 h to 0–10 U/ml rat thrombin (Sigma, T5772) in the presence or absence of selective inhibitor SCH79797 (R&D systems) and tcY-NH_2_ (TOCRIS), or agonist SFLLRN-NH_2_ (Sigma).

For knockdown of PAR1 or PAR3 expression in the astrocytes, the cells were cultured on the six-well plates for 24 h, followed by transfection of PAR1 siRNA3 (sense strand 5′-UCU UCA UAC CCU CCG UGU A dTdT-3′, antisense strand 5′-U ACA CGG AGG GUA UGA AGA dTdT-3′), PAR3 siRNA1 (sense strand 5′-CCA ACA UCA UAC UCA UAA U dTdT-3′, antisense strand 5′-U ACA CGG AGG GUA UGA AGA dTdT-3′), or scramble siRNA (sense strand 5′-GGC UCU AGA AAA GCC UAU GC dTdT-3′, antisense strand 5′-GC AUA GGC UUU UCU AGA GCC dTdT-3′) with iMAX transfection reagent (Invitrogen) for 36 h. The astrocytes were subsequently stimulated by 1 U/ml thrombin for another 2 h before morphological observation.

### Quantitative real-time polymerase chain reaction

Total RNA was extracted with Trizol (Sigma) from tissue samples of 1 cm spinal segments at lesion site, which were collected at 0 day, 1 day, 4 days, and 1 week following contusion (*n* = 6). The first-strand cDNA was synthesized using HiScript II Q RT SuperMix for qPCR (+gDNA wiper) (Vazyme) in a 20 µl reaction system, and diluted at 1:3 before used in assays. The primers were designed and synthesized by Invitrogen (Shanghai, China). The sequences of primers used in this study are as follows: for PAR1, forward primer 5′-ACT TCA CCT GCG TGG TCA TCT G -3′, reverse primer 5′-ATG GCG GAG AAG GCG GAG AA -3′; for GAPDH, forward primer 5′-GGG TCC CAG CTT AGG TTC AT-3′, reverse primer 5′-GAG GTC AAT GAA GGG GTC GT-3′. Reactions were carried out in a volume of 10 μl (1 μl cDNA template, 9 μl reaction buffer containing 5 μl of 2× ChamQ Universal SYBR qPCR Master Mix, 3 μl of RNase free ddH_2_O, and 0.5 μl of anti-sense and sense primers each). The qPCR assays were run with one initial denaturation cycle at 94 °C for 5 min, followed by 40 cycles of 94 °C for 30 s, 60 °C for 30 s, and 72 °C for 30 s. The expression levels were normalized to an endogenous *gapdh*. A negative control without the first-strand cDNA was also performed.

### Western blot

Total proteins were extracted from astrocytes or from tissue samples of 1 cm spinal segments at lesion sites following contusion at 0 day, 1 day, 4 days, and 1 week (*n* = 6). The extraction buffer contains 50 mM Tris (pH 7.4), 150 mM NaCl, 1% Triton X-100, 1% sodium deoxycholate, 0.1% SDS and 1 mM PMSF. Prior to the western blot analysis, the astrocytes were treated with 0–10 U/ml rat thrombin (sigma) or 100 μM PAR1 agonist SFLLRN-NH_2_ (Sigma) for 24 h. Samples were vortexed for 30 min, followed by a centrifugation at 12,000 rpm for 15 min. The supernatants were collected and protein concentrations were determined by the BCA method. Proteins of 20 μg of each sample were heated at 95 °C for 5 min, and then separated by 10% SDS-PAGE gel, followed by transferring onto a polyvinylidene difluoride (PVDF) membrane. The membrane was blocked with 5% skim milk in Tris-buffered saline containing 0.1% Tween-20 for 1 h, and then incubated with primary antibody overnight at 4 °C. The following primary antibodies were used in this study: p65NFκB (1:1000, CST); ERK (1:1000, CST); p-ERK (1:1000, CST); JNK (1:1000, CST); p-JNK (1:1000, CST); P38 (1:1000, CST); p-P38 (1:1000, CST). After washing three times with TBST (Tris buffered saline with Tween 20 containing 137 mM NaCl, 20 mM Tris and 0.1% Tween-20) for 10 min each, the membrane was incubated with secondary antibody goat-anti-mouse HRP or goat-anti-rabbit HRP (1:1000, Beyotime) for 2 h at room temperature. The blots were detected using an ECL kit, and the image was scanned by a GS800 Densitometer Scanner (Bio-Rad). Data were analyzed using PDQuest 7.2.0 software (Bio-Rad). The β-actin (1:5000) was used as an internal control.

### ELISA

Protein samples were prepared using the extraction buffer supplemented with a protease inhibitor PMSF as mentioned above. Homogenate was centrifuged at 12,000 rpm for 15 min at 4 °C, and the supernatant was collected for TNF-α, IL-1β, IL-6, IL-4, IL-10 (MULTI SCIENCES), and thrombin (Elabscience) ELISA assay. The contents of the cytokines were expressed as pg/ml/10^5^ cells for the supernatant, and pg/mg for the lysate of the cells and the cord tissues. Plates were read with a multifunctional enzyme marker (Biotek Synergy2) at a 450-nm wavelength.

### Tissue immunofluorescence

The segments of spinal cord were harvested, post-fixed in 4% paraformaldehyde for 4 h, and then stored overnight at 4 °C in 0.2 M phosphate buffer. The samples were subsequently cryoprotected in 30% sucrose for 48 h. Segments were embedded in OCT (VWR International), and frozen sectioned at 10 μm. After being blocked with 0.01 M PBS containing 3% BSA, 0.1% Triton X-100, and 10% normal goat serum for 1 h at 37 °C, the sections were incubated overnight at 4 °C with primary antibodies: GFAP (1:400, Sigma); PAR1 (1:400, Novusbio). Thereafter, the sections were washed with PBS and incubated with the Cy3-labeled goat anti-rabbit IgG (1:400, Abcam) or the Alexa Fluor 488-labeled donkey anti-mouse IgG (1:400, Abcam). Sections were photographed with fluorescence microscope (ZAISS, axio image M2).

### Cell viability assay

After cell treatment of 0–10 U/ml thrombin or 0–3 μM SCH79797, astrocytes were seeded into 96-well plates and culture for 24 h, followed by addition of 10 μl of 3-(4,5-dimethyl-2-thiazolyl)−2,5 -diphenyl-2-H-tetrazolium bromide (MTT; Sangon, Shanghai, China) for 4 h. Afterwards, dimethyl sulfoxide (DMSO; Aladdin, Shanghai, China) was added to dissolve formazan. The cell viability was evaluated by detecting absorbance at 570 nm with a Microplate Reader (BioTek, Synergy2).

### Cell proliferation assay

Primary astrocytes were collected and resuspended in fresh pre-warmed (37 °C) DMEM:F-12 medium at a density of 2 × 10^5^ cells/ml on 0.01% poly-l-lysine-coated 96-well plates. Following treatment with 1 U/ml thrombin or 100 μM SFLLRN-NH_2_ for 24 h, 50 mM EdU was added into the cultures and the plate was incubated for an additional 2 h to allow EdU incorporation. Subsequently, the cells were fixed with 4% formaldehyde in PBS for 30 min, and assayed using Cell-Light EdU DNA Cell Proliferation Kit (Ribobio). Astrocyte proliferation (ratio of EdU^+^ to all cells) was analyzed using images of randomly selected fields obtained on a DMR fluorescence microscope (Leica Microsystems, Bensheim, Germany). Assays were performed three times using triplicate wells.

### Cell migration assay

For the migration assay of astrocytes, the 6.5 mm transwell chambers with 8 μm pores (Corning Incorporated) were used as previously described [64]. Briefly, 100 μl of astrocytes (2 × 10^5^ cells/ml) was resuspended in DMEM and transferred to the top chambers of each transwell. Meanwhile, 1 U/ml thrombin or 100 μM SFLLRN-NH_2_ was added into the lower chambers. The cells were allowed to migrate at 37 °C in 5% CO_2_ for 24 h, and the upper surface of each membrane was cleaned with a cotton swab. Cells adhering to the bottom surface of each membrane were stained with 0.1% crystal violet, imaged, and counted using a DMR inverted microscope (Leica Microsystems). Assays were performed three times using triplicate wells.

### Statistical analysis

All data were presented as mean ± standard deviation (M ± SD). Statistical analysis was performed with GraphPad Prism 8 software (San Diego, CA, USA). Normality and homoscedasticity of the data were performed using Levene’s test. Independent sample *t*-test and One-way analysis of variance (ANOVA) followed by Bonferroni’s post-hoc comparisons tests were utilized for comparisons for different groups. Two-sided *p* value < 0.05 was accepted as statistically significant.

## Supplementary information

additional file 1

additional file 2

additional file 3

Original Data File

## Data Availability

The datasets used and/or analyzed during the current study are available from the corresponding author on reasonable request.
