# Supplementary material for: Thrombin induces morphological and inflammatory astrocytic responses via activation of PAR1 receptor
Source: Cell Death Discov. 2022 Apr 11;8:189. doi: 10.1038/s41420-022-00997-4 (PMC8995373; doi:10.1038/s41420-022-00997-4)
Supplement: Supplementary file 1 — additional file 1 [file 41420_2022_997_MOESM1_ESM.docx]

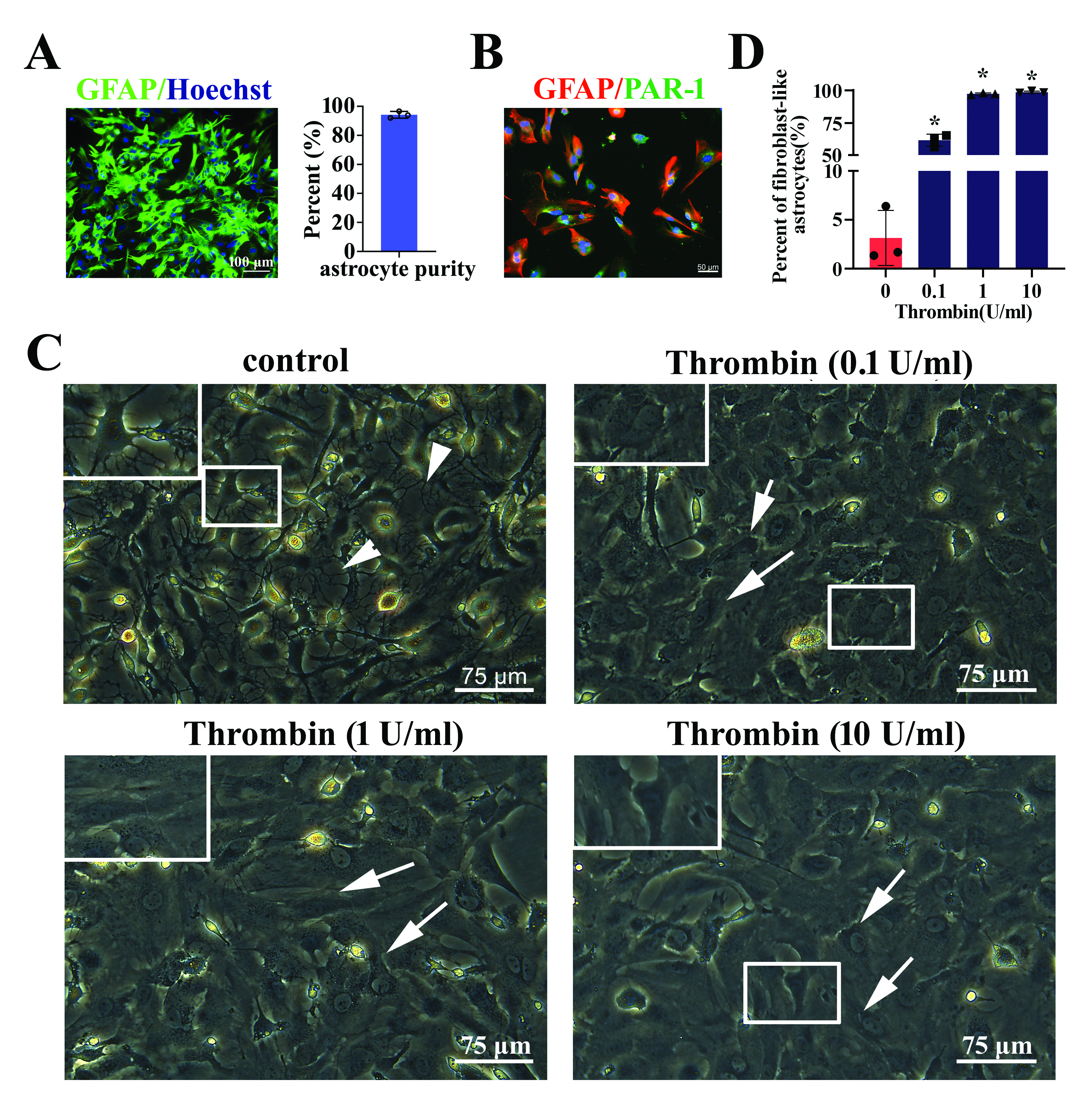


FigureS1. Primary culture of rat astrocytes and treatment with 0-10 U/ml thrombin. **A** Primary cultured rat astrocytes isolated from spinal cord stained with GFAP and Hoechst 33342 with purity over 95%. Experiments were performed in triplicates. Error bars represent the standard deviation (*P* < 0.05). **B** Immunostaining showed distribution of PAR1 receptor in the primary astrocytes. **C** Morphological changes of astrocytes following stimulation with 0-10 U/ml rat thrombin for 2 h. Arrowhead indicates stellate astrocyte, whereas arrow indicates fibroblast-like astrocyte. Rectangle indicates region magnified. **D** Statistical analysis of (C) in triplicates each 50 fields. Error bars represent the standard deviation (*P < 0.05). Scale bars, 100 μm in (A); 50 μm in (B); 75 μm in (C).
