# Supplementary material for: Thrombin induces morphological and inflammatory astrocytic responses via activation of PAR1 receptor
Source: Cell Death Discov. 2022 Apr 11;8:189. doi: 10.1038/s41420-022-00997-4 (PMC8995373; doi:10.1038/s41420-022-00997-4)
Supplement: Supplementary file 2 — additional file 2 [file 41420_2022_997_MOESM2_ESM.docx]

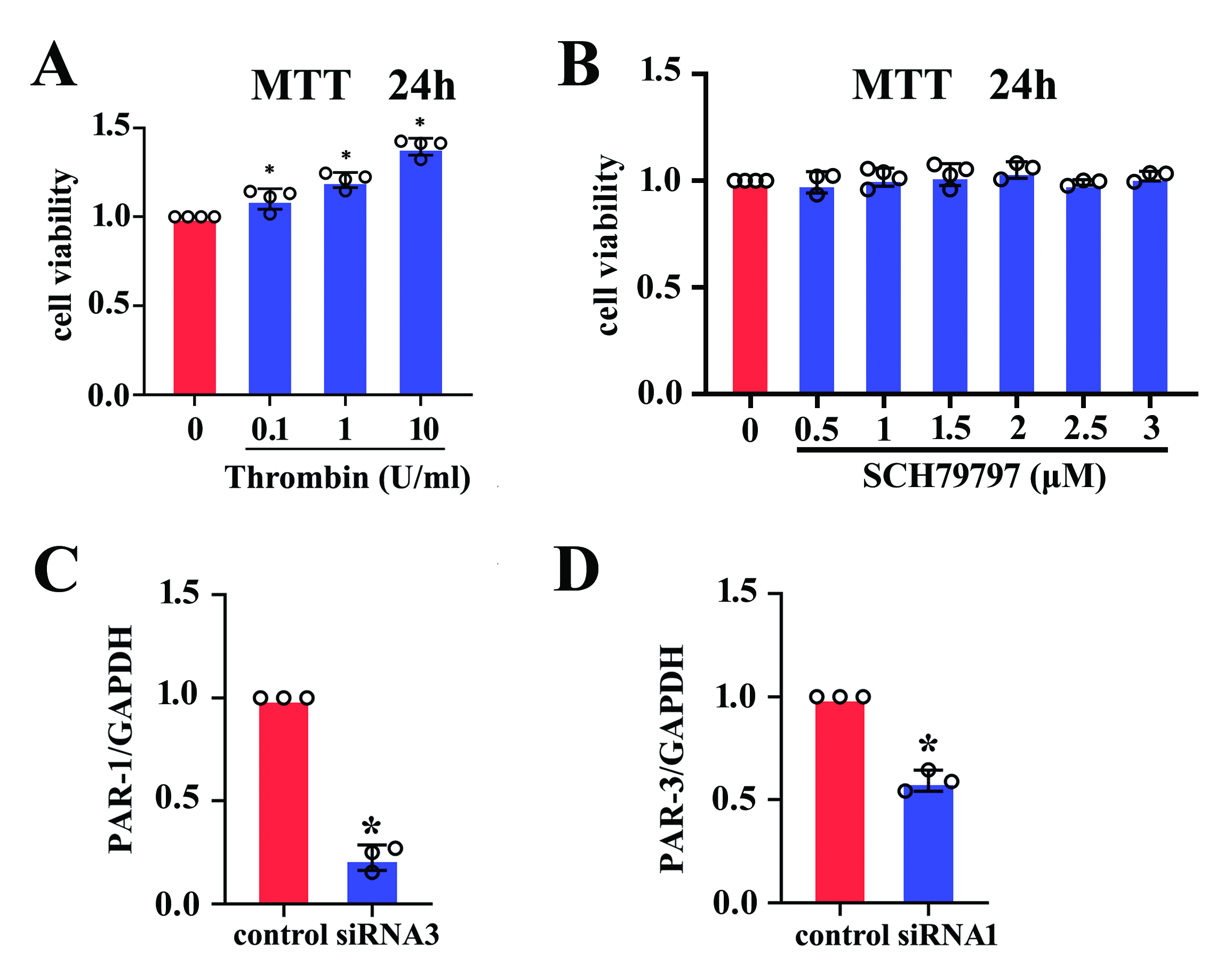


FigureS2. Determination of cell viability and interference efficiency of siRNA oligonucleotides. **A, B** MTT assay of cell viability following astrocyte treatment with various concentration of thrombin (A) and PAR1 antagonist SCH79797 (B). Experiments were performed in quadruplicates. Error bars represent the standard deviation (**P* < 0.05). **C, D** Interference efficiency of siRNA oligonucleotides for PAR1 (C) and PAR3 (D) at 36 h. Experiments were performed in triplicates. Error bars represent the standard deviation (**P* < 0.05).
