# Supplementary material for: Thrombin induces morphological and inflammatory astrocytic responses via activation of PAR1 receptor
Source: Cell Death Discov. 2022 Apr 11;8:189. doi: 10.1038/s41420-022-00997-4 (PMC8995373; doi:10.1038/s41420-022-00997-4)
Supplement: Supplementary file 3 — additional file 3 [file 41420_2022_997_MOESM3_ESM.docx]

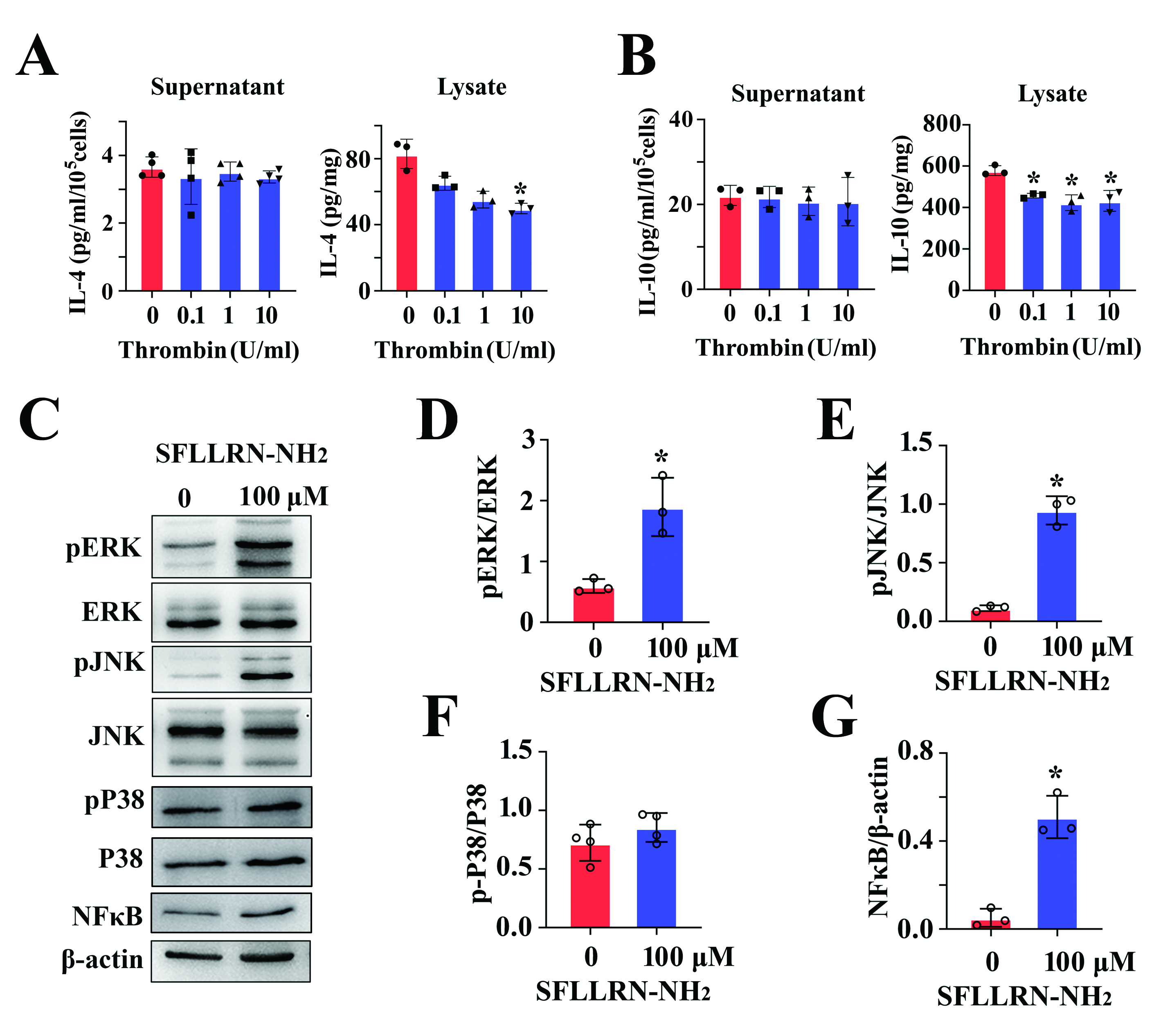


FigureS3. The effects of thrombin on the astrocyte production of ant-inflammatory cytokines and the PAR1 agonist SFLLRN-NH_2_ on the activation of MAPKs/NFκB signaling. **A, B** ELISA assay of IL-4 (A) and IL-10 (B) in the supernatant and lysate of astrocytes following stimulation with 0-10 U/ml thrombin for 24 h. Experiments were performed in triplicates. Error bars represent the standard deviation (*P < 0.05). **C** Western blot analysis of phosphorylation of ERK, P38, JNK kinase and NFκB protein after stimulation of astrocytes with 100 μM SFLLRN-NH_2_ for 24 h. **D-G** Quantification data as shown in (C). Quantities were normalized to endogenous β-actin. Experiments were performed in triplicates. Error bars represent the standard deviation (*P < 0.05).
