# Supplementary material for: Thrombin induces morphological and inflammatory astrocytic responses via activation of PAR1 receptor
Source: Cell Death Discov. 2022 Apr 11;8:189. doi: 10.1038/s41420-022-00997-4 (PMC8995373; doi:10.1038/s41420-022-00997-4)
Supplement: Supplementary file 4 — Original Data File [file 41420_2022_997_MOESM4_ESM.pdf]

Fig5.A

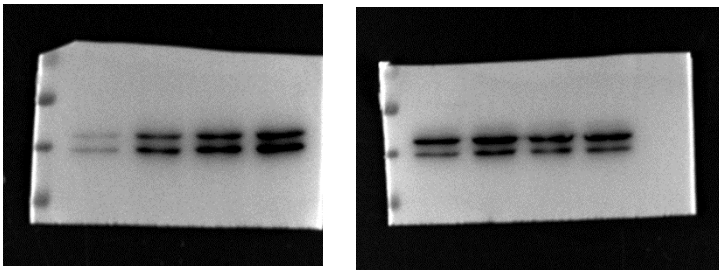

P- ERK ERK

Fig5.A

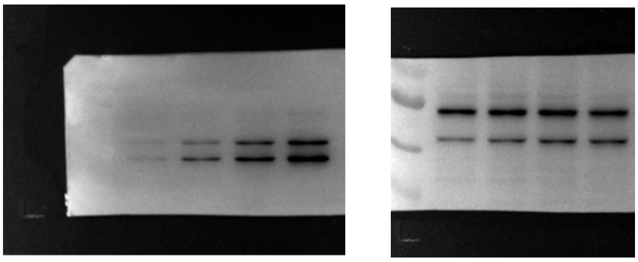

P-JNK JNK

Fig5.A

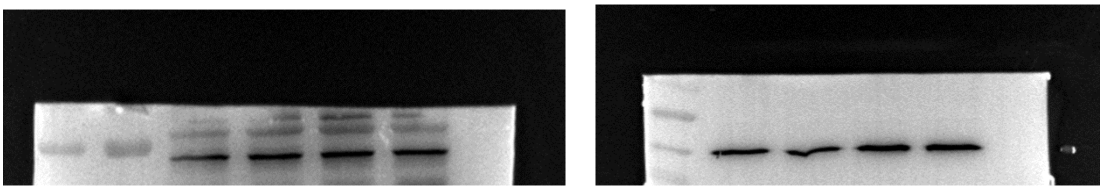

P-P38 P38

Fig5.A

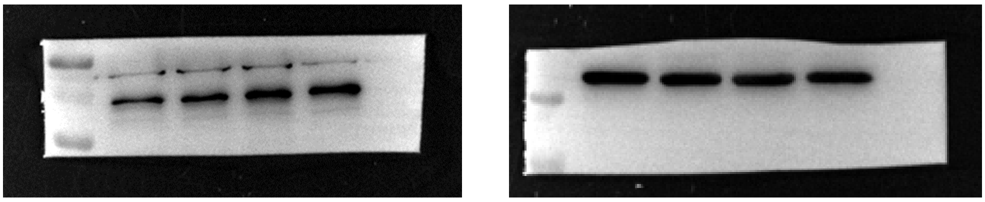

NF-κB β-actin

Fig6.A

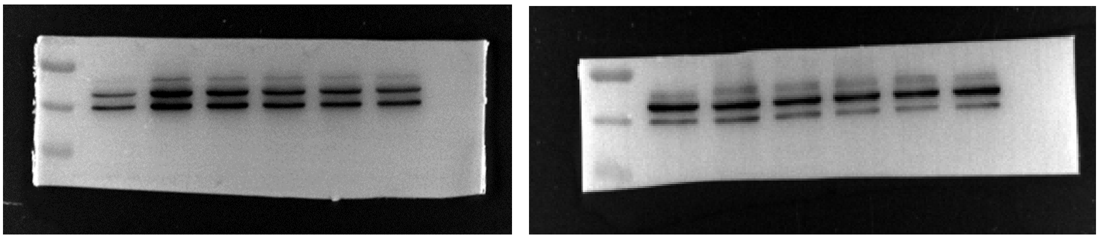

P- ERK ERK

Fig6.A

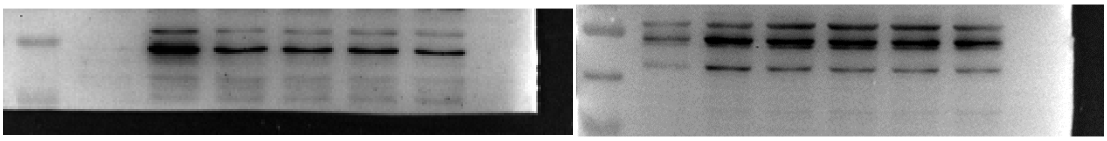

P-JNK JNK

Fig6.A

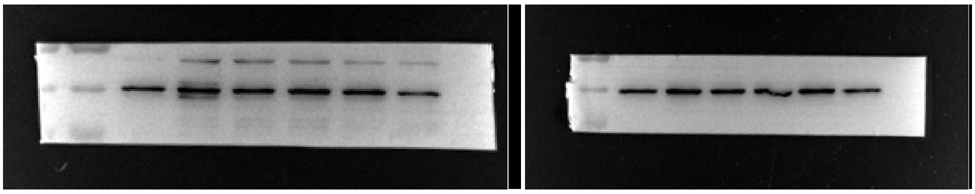

P-P38 P38

Fig6.A

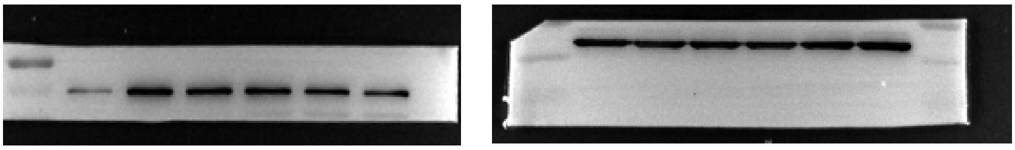

NF-κB β-actin

FigS3.C

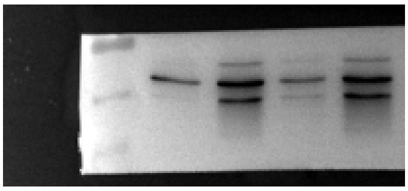

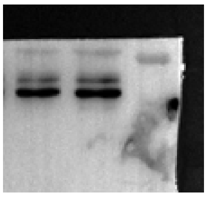

P- ERK

ERK

FigS3.C

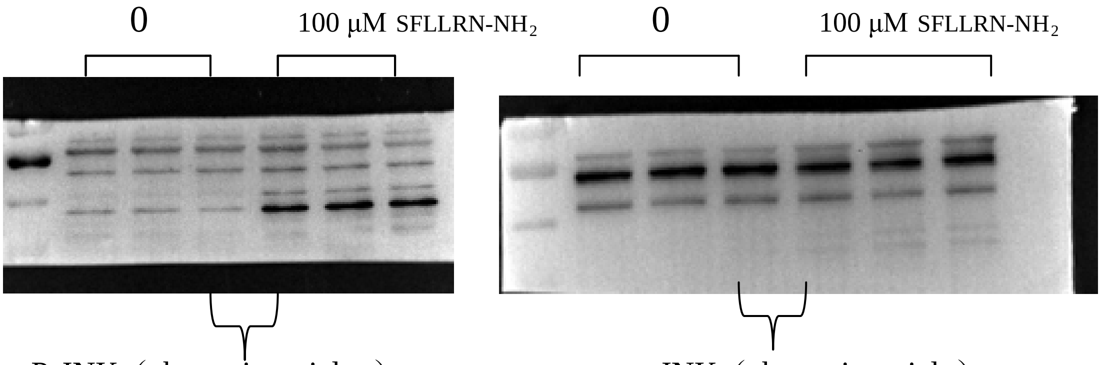

P-JNK（shown in article ）

JNK（shown in article）

FigS3.C

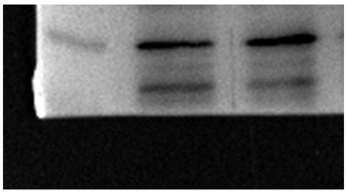

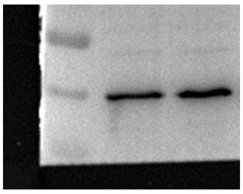

P-P38

P38

FigS3.C

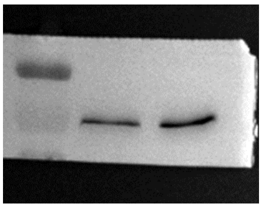

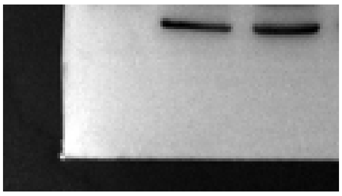

NF-κB

β-actin
